# A Study on the Laser-Assisted Machining of Carbon Fiber Reinforced Silicon Carbide

**DOI:** 10.3390/ma12132061

**Published:** 2019-06-27

**Authors:** Khulan Erdenechimeg, Ho-In Jeong, Choon-Man Lee

**Affiliations:** 1 Mechanical Design and Manufacturing, School of Mechatronics Engineering, Changwon National University, 20, Changwondaehak-ro, Uichang-gu, Changwon-si, Gyeongsangnam-do 51140, Korea; 2 Departement of Mechanical Engineering, College of Mechatronics, Changwon National University, 20, Changwondaehak-ro, Uichang-gu, Changwon-si, Gyeongsangnam-do 51140, Korea

**Keywords:** laser-assisted machining, Taguchi method, optimal machining conditions, machining characteristic

## Abstract

In recent years, as replacements for traditional manufacturing materials, monolithic ceramics and carbon fiber reinforced silicon carbide (C/SiC) ceramic matrix composites have seen significantly increased usage due to their superior characteristics of relatively low density, lightweight, and good high temperature mechanical properties. Demand for difficult-to-cut materials is increasing in a variety of area such as the automotive and aerospace industries, but these materials are inherently difficult to process because of their high hardness and brittleness. When difficult-to-cut materials are processed by conventional machining, tool life and quality are reduced due to the high cutting force and temperatures. Laser-assisted machining (LAM) is a method of cutting a workpiece by preheating with a laser heat source and lowering the strength of the material. LAM has been studied by many researchers, but studies on LAM of carbon–ceramic composites have been carried out by only a few researchers. This paper focuses on deducing the optimal machining parameters in the LAM of C/SiC. In this study, the Taguchi method is used to obtain the major parameters for the analysis of cutting force and surface roughness under various machining conditions. Before machining experiments, finite element analysis is performed to determine the effective depth of the cut. The cutting parameters for the LAM operation are the depth of cut, preheating temperature, feed rate, and spindle speed. The signal to noise (S/N) ratio and variance analysis (ANOVA) of the cutting force and surface roughness are analyzed, and the response optimization method is used to suggest the optimal machining parameters.

## 1. Introduction

**Figure 1:**
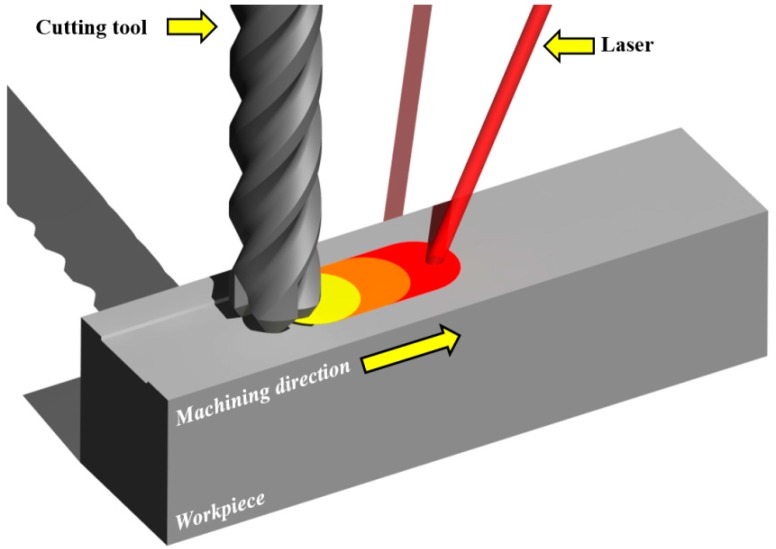
The schematic diagram of laser-assisted machining (LAM).

In recent years, due to their superior properties, carbon matrix ceramic composites have been increasingly utilized instead of monolithic ceramics and traditional manufacturing materials. Carbon matrix ceramic composites have low density, are lightweight, and have good high temperature strength. The demand for ceramic composite materials is growing in many fields such as, the aerospace and automobile industry, because of their high mechanical properties. However, the carbon matrix ceramic composites have high hardness, brittleness, are inhomogeneous and anisotropic in nature because its structure is composed of a brittle matrix and reinforcing fibers. The impact behaviors of the fibers, such as pullout and delamination, cause low surface quality after machining. The particles such as SiC and Al2O3 cause tool wear during machining [1,2,3,4,5]. Therefore, many researchers have studied the advanced machining technologies to machine the composite material effectively. The thermally assisted machining (TAM) is a method which is an effective process of machining difficult-to-cut materials [6]. TAM uses heat sources such as laser, induction, and plasma to locally preheat the workpiece and soften it. In particular, as laser technology has improved, many researchers have studied laser-assisted machining (LAM), a type of TAM [7,8]. The LAM is an eco-friendly machining method that uses the high-density laser beam to soften workpieces and removes the material with cutting tools along the machining path. In LAM, the thermal conductivity and specific heat of ceramic matrix composite is increased as the temperature is increased. When the C/SiC is preheated above 400 °C with a laser, the composite is oxidized. The material behavior is changed from brittle to ductile in the range of glass transition temperature (1050–1250 °C). When the oxidation rate is high, the coefficient of friction is decreased. When the coefficient of friction is decreased and material behavior is changed from brittle to ductile, material removal rate (MRR) and machinability are improved [9,10,11,12,13]. Figure 1 shows a schematic diagram of the LAM process.

Many researchers are still investigating efficient methods for processing ceramic composites materials. Many researchers have been actively studying the carbon fiber reinforced silicon carbide (C/SiC) composites and also have considered the structural integrity and reliability of high temperature structures such as exhaust valves, automobile parts, aircraft parts, and nozzle necks by composites [14,15,16]. Hui et al. investigated the changes in the tensile strength of C/SiC composites according to the changes of specimen cross-section and heat treatment conditions [17]. It was found that the fracture work and residual strain increased as the cross-section of the specimen decreased. Tao et al. conducted static and dynamic compression tests in various temperature ranges and studied the resulting changes in compressive strength [18]. Research has found that compressive strength increases with increasing temperature, but decreases with decreasing temperature. Fattahi et al. focused on the analytical prediction of delamination during drilling composite laminates [19]. Chi et al. investigated the effects of cylindrical specimen size for IG-110 and NBG-18 on the compressive strength and Weibull modulus [20]. Chinmaya et al. compared experimental and simulated results for cutting forces for machining of A359/SiC/20p composites [21]. The effects of operating conditions under LAM were determined for the shear zone stress of silicon nitride by experimentation. Shuting Lei et al. and Damian Przestackid focused on improving the Al/SiC composite machinability by LAM, and compared the results with those obtained when using the conventional turning process [22]. Except for the above-mentioned studies on the machining of composite materials using LAM, most studies have been performed with titanium and nickel alloys, and with various steels [23,24,25,26]. To date, there has been no research on using laser heat sources for preheating and machining of C/SiC composite materials. In this study, the response optimization method is used to determine the optimal machining parameters in LAM of C/SiC composite materials. This method is useful when evaluating the effect of multiple parameters on the response.

In this study, the experiments are performed to determine the optimal machining parameters and to analyze the thermal effect and machinability of the C/SiC composite under various machining conditions in the LAM process. The effective depth of cut for LAM of C/SiC composite is obtained by finite element analysis according to preheating temperature. Then, LAM experiments are performed for flat shaped C/SiC. The influence of the machining parameters such as depth of cut, preheating temperature, feed rate, and spindle speed on cutting force, surface roughness, and tool wear is analyzed using the Taguchi method. The cutting force is measured by a dynamometer and the surface roughness is measured by the shape measuring device. For the determination of the optimal machining parameters, the response optimization method is performed. An efficient machining condition to increase machinability is proposed and discussed.

## 2. Finite Element Analysis

### 2.1. Finite Element Analysis

**Figure 2:**
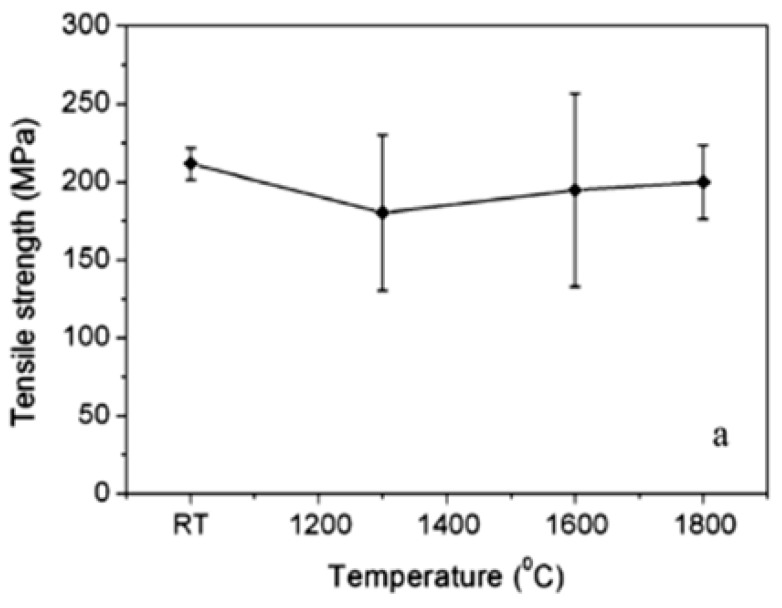
The tensile stress value according to temperature of C/SiC composite.

**Figure 3:**
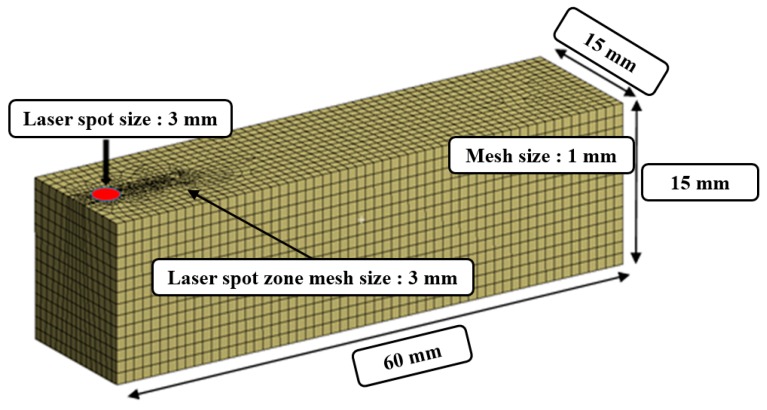
The finite element analysis model.

The finite element analysis method is performed to study the overlapping in the laser heat source according to the feed rate. The moving time of the heat source for the analysis was set to be in the range of 10–30 sec by considering the feed rate (f = 100–300 mm/min). The material properties for the analysis are listed in Table 1. The main components of C/SiC composite are reported in Table 2. The preheating temperature was chosen by considering the tensile strength of C/SiC composite material according to the temperature, as shown in Figure 2. The tensile strength of the C/SiC composite decreases at the temperature ranges from the room temperature to 1300 °C [27]. Additionally, C/SiC composite material has its maximum elongation and minimum tensile strength at a temperature of about 1300 °C. The temperature range of the effective depth of the cut is 1100–1300 °C. To increase the accuracy of the analysis, the mesh elements are divided into squares of 1 mm and the preheated sections are localized at a mesh size of 0.5 mm. The mesh consists of 66,267 nodes and 16,425 elements. Figure 3 shows the analysis model of the specimen and shows the shape of the generated mesh.

### 2.2. Result of Analyis

**Figure 4:**
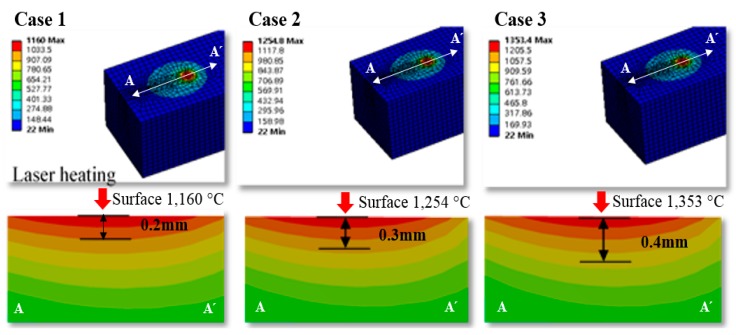
The finite element analysis result and the effective depth of the cut according to the preheating temperature.

The finite element analysis result, the temperature distribution for the section view of the workpiece was used to determine the effective depth of cut according to preheating temperature. The effective depth of the cut is selected to be the depth at the temperature range of 1100–1300 °C where the strength of the material decreases. The analysis result, the effective depth of the cut was determined to be in the region of 0.2–0.4 mm at a preheating temperature range of 1100–1300 °C. Figure 4 shows the finite element analysis results, and effective depth of cut according to the preheating temperature.

## 3. Laser-Assisted Machining

### 3.1. Procedure

**Figure 5:**
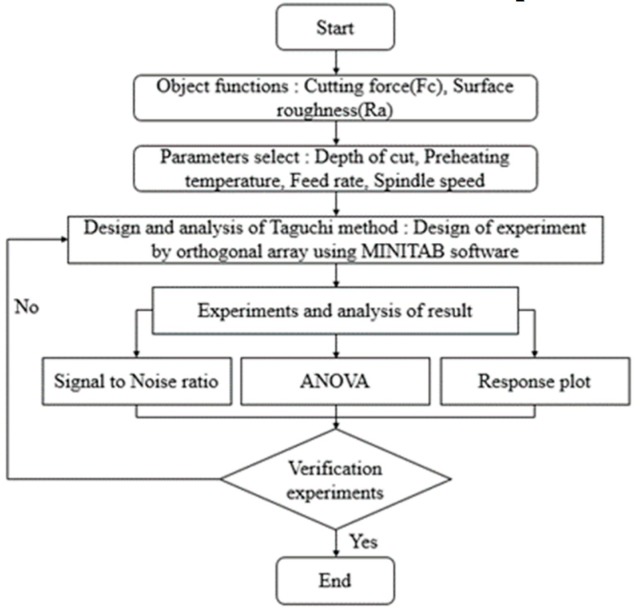
Flow chart of the design of experiments for the laser-assisted machining (LAM) of the C/SiC composite.

The design of the experiment was performed to determine optimal machining conditions for the LAM of C/SiC composites. The object functions are selected as cutting force and surface roughness. The parameters such as depth of cut, preheating temperature, spindle speed, and feed rate are selected. The depth of cut is determined by the results of finite element analysis. Figure 5 shows the flow chart of the design of experiments for the LAM of C/SiC composite.

### 3.2. Machining Conditions

**Figure 6:**
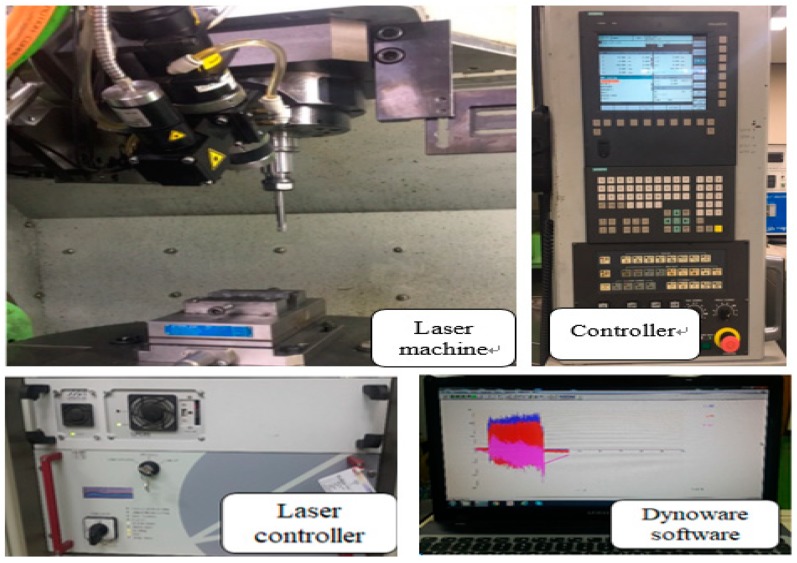
The experimental setup.

The LAM process was performed on the 5-axis machining center (Hyundai WIA., Type Hi-V560M) with laser module. The laser module is a high-power diode laser (HPDL) with a wavelength range of 940–980 nm (Laser line Inc., Type LDM 1000-100). To measure the preheating temperature, a pyrometer (Dr. Mergemthaler GmbH & Co. KG, LPC03) with a range of 400–3000 °C was used. The dynamometer (Kistler Inc., 9257B) attached to the indexing table was used to measure the cutting forces. A dynamometer measures the three orthogonal components of a force using the quartz three-component measurement. The measurement range is −5 kN to 5 kN, and the rigidity is 1 kN/μm to 2 kN/μm. The surface roughness measurement device (Kosaka Inc., SE-3500K) and field emission scanning electron microscope (ZEISS Inc., MERLIN) were used to measure the surface roughness. The surface roughness measurement device was a probe type with a resolution of 32,000 points/16 bite. The surface roughness measurement device used the Gaussian profile filter to separate the long and short wave of a surface profile, and a cut-off value of 0.25 mm was used in this study. Figure 6 shows the experimental set-up and Table 3 shows machining conditions.

### 3.3. Experimental Design

In LAM, machining characteristics of C/SiC composite are affected by various factors. To analyze the influence of the factors on the machining characteristics, the experiments should be performed after considering all the combinations of factors. However, in this case, the number of experiments increases and it is costly and time-consuming. Therefore, the design of experiment should be performed to reduce the cost and time. The experimental design proposed by the Taguchi method uses orthogonal arrays to organize the parameters that affect the process and varies the levels of those parameters. The Taguchi approach values the importance of the logic of the parameters and has a strong effect compared to actual experiments [28,29,30,31,32]. The Taguchi method used signal to noise (S/N) as a quality characteristic. The S/N ratio characteristics can be divided into three types: nominal-is-best characteristics, larger-the-better characteristics, and smaller-the-better characteristics. In this study, the smaller-the-better characteristics were used. The smaller-the-better characteristics are shown in Equation (1).

(1) $$\mathrm{SN}=-10\log[\frac{1}{n}\sum_{i=1}^{n}y_{i}^{2}]$$

where, *y_i_* is the average of the observed data, *n* is the number of observations, and *y* represents the observed data or each type of characteristic; with the above S/N ratio transformation, the smaller the S/N ratio is, the better are the results for cutting force and surface roughness.

The cutting parameters for the LAM are the depth of cut (A), preheating temperature (B), feed rate (C), and spindle speed (D). The depth of the cut range (0.2–0.4 mm) was selected based on the finite element analysis result. The preheating temperature range (1100–1300 °C) was selected based on the tensile strength of C/SiC composite material according to the temperature. The feed rate range (100–300 mm/min) and the spindle speed range (2000–8000 rpm) were selected after considering previous studies. The conventional machining (CM) was performed to verify the efficiency of the LAM. Table 4 shows the factors and levels used in the experiments and Table 5 shows the experimental layout using an L_9_ orthogonal array [33,34].

### 3.4. Experimental Results on LAM

**Figure 7:**
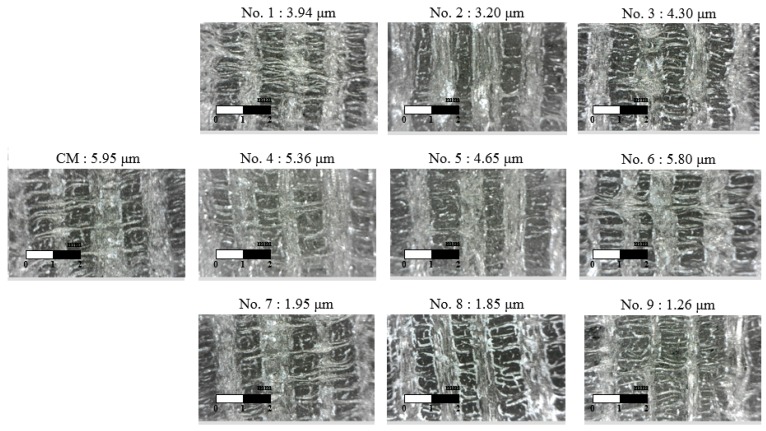
The microphotographs of machined surfaces of C/SiC composite material in LAM.

**Figure 8:**
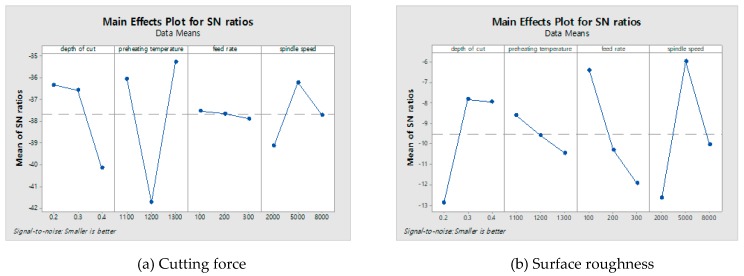
The main effect plot of C/SiC composite on the cutting force and the surface roughness. (**a**) Cutting force; (**b**) Surface roughness.

The cutting forces were measured using a tool dynamometer during the machining of C/SiC composite material. The surface integrity was analyzed by the surface roughness measurement device and a field emission scanning electron microscope (FE-SEM). All experiments were repeated three times. The cutting force was calculated by the average value of each experiment and the surface roughness was used as the lowest value of each experiment. Figure 7 shows the microphotographs of the machined surfaces of C/SiC composite material in all experiments. Table 6 shows the measured cutting force and surface roughness according to the four factors of the machining conditions. In LAM, the cutting force was decreased by about 40.7% and the surface roughness was decreased by about 33.8%, compare to the CM. The lowest cutting force value at A_2_B_3_C_1_D_2_ was 42.25 N, and the surface roughness of the S/N ratio had the highest value of −32.5165 dB. Figure 8a shows that the optimal levels were found to be A_2_ (depth of cut: 0.3 mm), B_3_ (preheating temperature: 1300 °C), C_1_ (feed rate: 100 mm/min), and D_2_ (spindle speed: 5000 rpm). The lowest surface roughness value at A_2_B_3_C_1_D_2_ was 1.26 μm, and the surface roughness of the S/N ratio had the highest value of −2.0074 dB. Figure 8b shows the optimal levels were found to be A_2_ (depth of cut: 0.3 mm), B_3_ (preheating temperature: 1300 °C), C_1_ (feed rate: 100 mm/min), and D_2_ (spindle speed: 5000 rpm). Table 7 and Table 8 shows the response table mean S/N ratio for the cutting force and surface roughness according to the machining conditions.

### 3.5. Variance Analysis

Variance analysis (ANOVA) was applied to the S/N ratios to determine the relations between machining parameters relating to surface roughness and cutting force. The calculated S/N ratio for the four factors of the surface roughness and cutting force in the machining of C/SiC composites are shown in Table 9 and Table 10. In these results, the most significant influences on the cutting force value for each factor were the percentage contributions of the factors of preheating temperature, depth of cut, spindle speed, and feed rate; these percentages were 66.23%, 22.55%, 9.91%, and 1.31%, respectively. Also, the factors that contributed to the surface roughness were determined; the most important factor was 31.24% for the spindle speed, the second factor was 29.69% for the depth of cut, the third factor was 27. 93% for the preheating temperature, and the fourth factor was 11.13% for the feed rate.

## 4. Experimental Results and Discussion

### 4.1. Signal to Noise (S/N) Ratio of Analysis

S/N ratio is a very important measurement in the Taguchi method for experimental data analysis. According to the Taguchi approach, optimal machining condition values should lead to a maximum S/N ratio. Parameter values are important factors for evaluating the surface roughness and cutting force. Other characteristics contribute slightly to the cutting force and surface roughness evaluation. Results of machining experiments have been studied using the S/N ratio. Based on the predictions and response results of the ANOVA analyses, optimal machining parameters for cutting force and surface roughness were obtained and verified. The correlation test between the cutting force and surface roughness was performed by analyzing the correlation coefficient (r). The result of the correlation test, the correlation between the two parameters was a positive correlation and r was 0.223. The correlation coefficient ranged from −1 to 1 and describes the parametric value of linear relationship.

### 4.2. Response Optimization

The objective of this experiment is to optimize the machining parameters and to develop better (i.e., low value) surface roughness and cutting force values; the “smaller the better” characteristic was used. The optimal machining conditions, which were the depth of cut of 0.3 mm, preheating temperature of 1100 °C, the feed rate of 200 mm/min, and a spindle speed of 5000 rpm were obtained for the best cutting force and surface roughness values. The desirability is confirmed at 1. According to the Taguchi design results obtained for the cutting force and surface roughness, the response optimization results are given in Table 11 and Table 12.

### 4.3. Prediction Equations and Confirmation Experiments of the Optimal Condition

Confirmation experiments were conducted to calculate the suitability of the analysis results. The prediction equations for the cutting force and the surface roughness are shown in Equations (2) and (3).

*Fc* = 83.48 + 10.83 (S2000) − 15.94 (S5000) + 5.108 (S8000) + 4.661 (F100) − 5.476 (F200) + 0.8144 (F300) − 19.82 (P1100) + 42.07 (P1200) − 22.25 (P1300) − 16.36 (D0.2) − 7.692 (D0.3) + 24.05 (0.4) (2)

**Figure 9:**
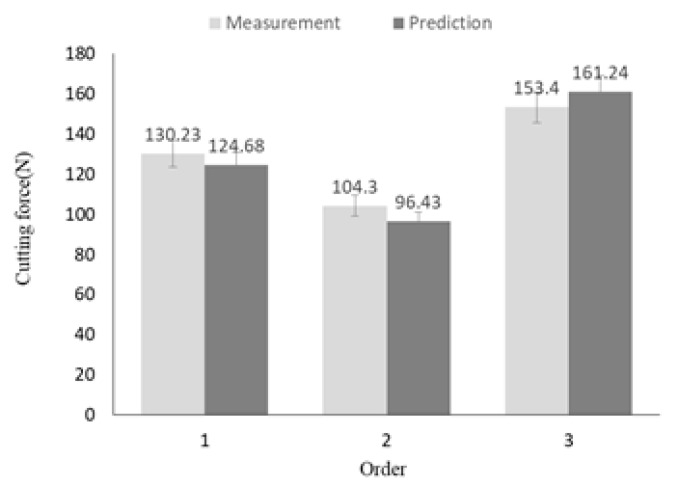
The comparison of the cutting force between the prediction equation results and the confirmation experiment results.

**Figure 10:**
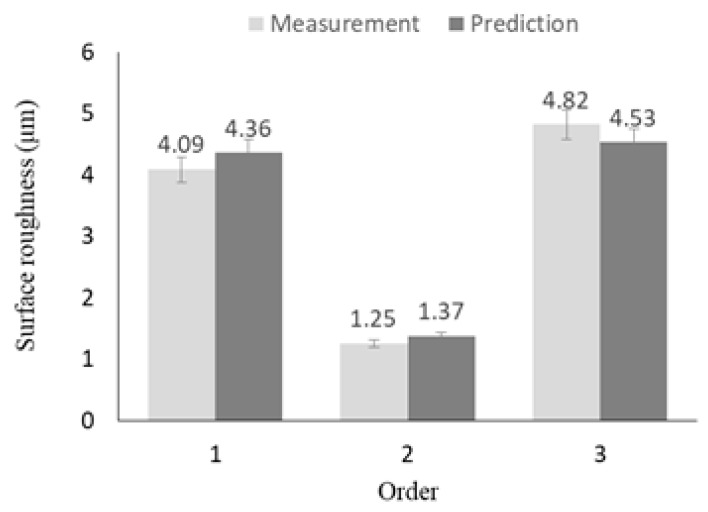
The comparison of the surface roughness between the prediction equation results and the confirmation experiment results.

*Ra* = 3.388 + 0.9089 (S2000) − 1.251 (S5000) + 0.3422 (S8000) − 1.038 (F100) − 0.04111 (F200) + 1.079 (F300) − 0.5778 (P1100) − 0.1544 (P1200) + 0.7322 (P1300) + 1.259 (D0.2) − 0.5711 (D0.3) − 0.6878 (D0.4) (3)

where, *Fc* represents the cutting force, and *Ra* represents the surface roughness. Table 13 shows the machining conditions of the confirmation experiment. The three experiments were conducted by randomly adding four machining conditions, including optimal machining conditions (Exp. No. 1) and the main effect of surface roughness (Exp. No. 2), are deducted in Table 13. All experiments were repeated three times. Figure 9 shows the comparison of the results of the prediction equation and the confirmation experiments for the cutting force. As a result of the comparison, the maximum error rate was confirmed to be approximately 7.55%. Figure 10 shows the comparison of the results of the prediction equation and the confirmation experiments for the surface roughness. As a result of the comparison, the maximum error rate was confirmed to be approximately 8.76%. Confirmation experiments were conducted to verify the optimal machining parameters.

## 5. Conclusions

In this study, LAM was carried out on the C/SiC composite material. The effective depth of cut was selected using the finite element analysis. The optimal machining conditions were obtained using the Taguchi method, which uses cutting force and surface roughness as objective function. The conclusions obtained from this study are as follows.

(1). The finite element analysis was performed to determine the preheating temperature and the depth of cut depending on the tensile strength of the C/SiC composite material. When the preheating temperature is in the tensile strength decreasing range (1100–1300 °C), the effective depth of cut is determined to be in the range of 0.2–0.4 mm.
(2). According to the Taguchi standard design concept in this experiment, at three levels with four factors of each one, nine experiments must be performed, and fractional design was selected in a standard L9 orthogonal array. The maximum value was found using the S/N ratio equation of “the smaller-the better”; the maximum S/N ratio yielded the optimal machining parameters.
(3). In same case of the machining conditions, the cutting force was decreased by about 40.7% compared to CM in LAM of the C/SiC composite material, and the surface roughness was decreased by about 33.8% compared to CM in LAM of the C/SiC composite material.
(4). Variance analysis (ANOVA) was applied to the S/N ratio to discover the interactions between the parameters relating to surface roughness (Ra) and cutting force (Fc). Based on the ANOVA results, the main contributing factor for the cutting force was 66.23% preheating temperature. The main contributing factor for the surface roughness was 31.24% spindle speed.
(5). The verification experiment was performed to construct the predictive equation and to ensure the reliability of the predictive equation. The verification experiment confirmed that the maximum error was 7.55% between the prediction equation for cutting force and measurement experiment value. The maximum error was 8.76% between the prediction equation for surface roughness and measurement experiment value. The prediction equation demonstrated the reliability of low error.

The results of response optimization, the optimal machining conditions for LAM of the C/SiC composite material were obtained as spindle speed (5000 rpm), feed rate (200 mm/min), preheating temperature (1100 °C), and DOC (0.3 mm). When the experiment was performed by optimal machining conditions, the cutting force was measured to be 34.55 N and surface roughness was measured to be 0.95 µm.

## Figures and Tables

**Table 1:** Mechanical properties of C/SiC composite.

Density (g/cm^3^)	Young Modulus (GPa)	Thermal Conductivity (W/mm-K)	Specific Heating (J/kg-K)	Flexural Strength (MPa)
2.1	35	40	1200	67

**Table 2:** The main components of C/SiC composite.

The Composition by X-ray Analysis (%)			Open Porosity (qv) (%)
C	SiC	Residual Si	
50.47	44.81	4.72	5.40

**Table 3:** The machining conditions.

Material	C/SiC Composite
Material size (T × W × L, mm)	15 × 15 × 60
Machining method	Slot milling
Cutting tool	D8 CBN flat end-mill, 2F, 70L

**Table 4:** The factors and levels used in the experiments.

Symbol	Factor	Level 1	Level 2	Level 3
A	Depth of cut (mm)	0.2	0.3	0.4
B	Preheating temperature (°C)	1100	1200	1300
C	Feed rate (mm/min)	100	200	300
D	Spindle speed (rpm)	2000	5000	8000

**Table 5:** The experimental layout using an L_9_ orthogonal array.

Experiment No.	Depth of Cut (mm)	Preheating Temperature (°C)	Feed Rate (mm/min)	Spindle Speed (rpm)
CM	0.2	1100	100	2000
1	0.2	1100	100	2000
2	0.2	1200	200	5000
3	0.2	1300	300	8000
4	0.3	1100	200	8000
5	0.3	1200	300	2000
6	0.3	1300	100	5000
7	0.4	1100	300	5000
8	0.4	1200	100	8000
9	0.4	1300	200	2000

**Table 6:** The experimental data value of cutting force and surface roughness.

No.	Depth of Cut (mm)	Preheating Temperature (°C)	Feed Rate (mm/min)	Spindle Speed (rpm)	Surface Roughness (μm)	Cutting Force (N)
CM	0.2	1100	100	2000	5.95	105.90
1	0.2	1100	100	2000	3.94	62.80
2	0.2	1200	200	5000	3.20	87.77
3	0.2	1300	300	8000	6.80	50.79
4	0.3	1100	200	8000	2.54	55.60
5	0.3	1200	300	2000	4.65	129.50
6	0.3	1300	100	5000	1.26	42.25
7	0.4	1100	300	5000	1.95	72.58
8	0.4	1200	100	8000	1.85	159.36
9	0.4	1300	200	2000	4.30	90.63

**Table 7:** The response table mean signal to noise (S/N) ratio for the cutting force according to the machining conditions.

Level	Depth of Cut (A)	Preheating Temperature (B)	Feed Rate (C)	Spindle Speed (D)
1	−36.31	−36.03	−37.51	−39.12
2	−36.55	−41.72	−37.64	−36.20
3	−40.14	−35.26	−37.86	−37.69
Delta	3.82	6.46	0.35	2.92
Rank	2	1	4	3

**Table 8:** The response table mean S/N ratio for the surface roughness according to the machining conditions.

Level	Depth of Cut (A)	Preheating Temperature (B)	Feed Rate (C)	Spindle Speed (D)
1	−12.888	−8.602	−6.420	−12.643
2	−7.818	−9.598	−10.290	−5.970
3	−7.938	−10.442	−11.933	−10.030
Delta	5.070	1.840	5.513	6.672
Rank	3	4	2	1

**Table 9:** The analysis results of variance for cutting force.

Factors	Degree of Freedom	Sum of Squares	Mean of Squares	Contribution (%)
Feed rate	2	157.1	78.56	1.31%
Spindle speed	2	1192.9	596.44	9.91%
Depth of cut	2	2714.9	1357.46	22.55%
Preheating temperature	2	7972.5	3986.27	66.23%
Error	0	*	*	*
Total	8	12037.5	-	100

**Table 10:** The analysis results of variance for surface roughness.

Factors	Degree of Freedom	Sum of Squares	Mean of Squares	Contribution (%)
Feed rate	2	6.728	3.364	27.93%
Spindle speed	2	7.525	3.763	31.24%
Depth of cut	2	7.152	3.576	29.69%
Preheating temperature	2	2.681	1.341	11.13%
Error	0	*	*	*
Total	8	24.087	-	100

**Table 11:** The response optimization.

Parameter	Goal	Target	Upper	Weight	Importance
Cutting force	Minimum	42.25	159.36	1	1
Surface roughness	Minimum	1.26	6.80	1	1

**Table 12:** Response optimization results.

Depth of Cut (mm)	0.3
Preheat temperature (°C)	1100
Feed rate (mm/min)	200
Spindle speed (rpm)	5000
Cutting force optimization plot (N)	34.55
Surface roughness optimization plot (µm)	0.946667
Desirability	1

**Table 13:** The machining conditions for confirmation experiments.

Exp. No.	Depth of Cut (mm)	Preheating Temperature (°C)	Feed Rate (mm/min)	Spindle Speed (rpm)
1	0.2	1200	100	2000
2	0.3	1200	200	5000
3	0.4	1200	300	2000
